# 183例伴SF3B1基因突变髓系肿瘤患者的突变特征及预后

**DOI:** 10.3760/cma.j.cn121090-20260303-00116

**Published:** 2026-08

**Authors:** 慧敏 张, 妮 武, 艾蕊 江, 珺丹 解, 红 姚, 谦 王, 丹丹 刘, 金兰 潘, 子兴 陈, 苏宁 陈, 宏杰 沈

**Affiliations:** 苏州大学附属第一医院，国家血液系统疾病临床医学研究中心，江苏省血液研究所，国家卫生健康委员会血栓与止血重点实验室，苏州 215006 The First Affiliated Hospital of Soochow University, National Clinical Research Center for Hematologic Diseases, Jiangsu Institute of Hematology, National Health Commission Key Laboratory of Thrombosis and Hemostasis, Suzhou 215006, China

**Keywords:** 血液肿瘤, 髓系, SF3B1, 突变, Hematologic neoplasms, Myeloid, SF3B1, Mutation

## Abstract

**目的:**

分析伴SF3B1突变髓系肿瘤患者的突变特征及预后。

**方法:**

本回顾性队列研究收集2016年10月至2024年12月苏州大学附属第一医院诊治的183例伴SF3B1突变的髓系肿瘤患者的临床资料，采用高通量测序检测髓系肿瘤常见52种基因突变，分析患者的突变特征及预后。

**结果:**

在本研究病例中，共计检测到31种SF3B1突变类型，突变位点主要分布在SF3B1基因HEAT结构域第581～933位氨基酸。骨髓增生异常肿瘤（MDS）患者中位基因突变数［2（1～7）］低于伴SF3B1突变的骨髓增生异常肿瘤/骨髓增殖性肿瘤（MDS/MPN）患者［4（1～9）］和急性髓系白血病（AML）患者［3（1～9）］，差异均有统计学意义（*P*值均<0.001）。在克隆性造血和原始细胞比例<5％的MDS患者中，SF3B1突变变异等位基因频率（VAF）<10％的患者中位HGB水平高于SF3B1突变VAF≥10％的患者（95 g/L对76 g/L，*P*＝0.002）。并且，SF3B1突变VAF<10％的患者中位环状铁粒幼红细胞比例低于SF3B1突变VAF≥10％的患者（3％对41％，*P*<0.001）。此外，MDS/MPN患者中JAK2 V617F突变和RUNX1突变的发生率高于MDS患者（22.2％对1.2％，*P*＝0.001；33.3％对6.6％，*P*＝0.002）。AML患者中RUNX1和RAS通路相关基因突变发生率均高于MDS患者（24.2％对6.6％，*P*＝0.008；66.7％对3.8％，*P*<0.001）。在MDS和MDS/MPN中，RUNX1突变阳性患者较RUNX1突变野生型患者具有更高的AML进展风险（*P*<0.001）。MDS-SF3B1（91.6％）和MDS/MPN（79.9％）患者的5年总生存率优于AML（63.8％）患者（*P*值分别为<0.001和0.033）。

**结论:**

SF3B1突变髓系肿瘤不同亚型患者具有不同的突变谱及预后。

## Abstract (en)

**Objective:**

To analyze the mutational characteristics and prognosis of patients with myeloid neoplasms and SF3B1 mutations.

**Methods:**

This retrospective cohort study analyzed clinical data from 183 patients with myeloid neoplasms and SF3B1 mutations diagnosed at the First Affiliated Hospital of Soochow University from October 2016 to December 2024. High-throughput sequencing was used to detect mutations in 52 common genes associated with myeloid neoplasms, and the patients' mutational characteristics were examined.

**Results:**

A total of 31 types of SF3B1 mutations were detected in the present study. SF3B1 mutations mainly occurred within the HEAT domain, spanning amino acids 581 to 933. The median number of gene mutations in patients with myelodysplastic neoplasms（MDS）［2（1–7）］was lower than that in patients with SF3B1-mutated myelodysplastic/myeloproliferative neoplasms（MDS/MPN）［4（1–9）］and acute myeloid leukemia（AML）［3（1–9）］, with all differences being significant（all *P*<0.001）. Among patients with clonal hematopoiesis and MDS with <5％ blasts, those with an SF3B1 mutational variant allele frequency（VAF）of <10％ had higher median HGB levels than those with an SF3B1 mutation VAF of ≥10％（95 g/L *vs* 76 g/L, *P*＝0.002）. Patients with an SF3B1 mutation VAF of <10％ had a lower median percentage of ring sideroblasts than those with an SF3B1 mutation VAF of ≥10％（3％ *vs* 41％, *P*<0.001）. Furthermore, the incidence of JAK2 V617F mutations was higher in the MDS/MPN patients than in the MDS patients（22.2％ *vs* 1.2％, *P*＝0.001）, as was the incidence of RUNX1 mutations（33.3％ *vs* 6.6％, *P*＝0.002）. The incidence of RUNX1 mutation was higher in AML patients than in MDS patients（24.2％ *vs* 6.6％, *P*＝0.008）, as was the RAS family gene mutations（66.7％ *vs* 3.8％, *P*<0.001）. In MDS and MDS/MPN, patients with RUNX1 mutations had a higher risk of progression to AML than those without such a mutation（*P*<0.001）. Patients with MDS-SF3B1（91.6％）and MDS/MPN（79.9％）demonstrated better 5-year overall survival than those with AML（63.8％）（*P*< 0.001 and *P*＝0.033, respectively).

**Conclusion:**

Patients with different subtypes of SF3B1-mutated myeloid neoplasms exhibit distinct mutational profiles and prognoses.

世界卫生组织（World Health Organization，WHO）第5版造血和淋巴组织肿瘤分型指南在骨髓增生异常肿瘤（myelodysplastic neoplasms，MDS）中新增MDS伴低原始细胞和SF3B1突变（MDS-SF3B1）亚型[1]。SF3B1基因位于染色体2q33.1，编码剪接因子3b蛋白复合物的亚基1。SF3B1基因编码的蛋白主要通过C端22个非同源的串联HEAT重复结构域调控基因剪接，参与肿瘤细胞发生发展[2]。SF3B1突变可见于克隆性造血（clonal haematopoiesis，CH）、骨髓增殖性肿瘤（myeloproliferative neoplasms，MPN）、MDS、MDS/MPN、急性髓系白血病（acute myeloid leukemia，AML）等髓系肿瘤[3]–[7]。CH具有向MDS、AML进展的风险[8]。为研究伴SF3B1突变髓系肿瘤患者的突变特征，本研究回顾性分析在苏州大学附属第一医院确诊的183例伴SF3B1突变髓系肿瘤患者的突变特征、临床特征及预后，为临床诊断提供参考。

## 病例与方法

1. 病例：本项回顾性队列分析纳入苏州大学附属第一医院2016年10月至2024年12月诊断的183例SF3B1突变髓系肿瘤患者，根据WHO第5版造血和淋巴组织肿瘤分型指南标准进行诊断分型[1]。本研究已通过苏州大学附属第一医院伦理委员会批准（批准号：2025伦审批第815号），并获得患者的知情同意。

2. 染色体核型分析：取患者肝素抗凝骨髓标本，以2×10^6^/ml的细胞密度接种于含20％小牛血清（美国Gibco公司产品）的RPMI 1640培养基中，培养24 h，于收获前1 h加入秋水仙酰胺（美国Sigma公司产品），终浓度为0.2 µg/ml，将细胞阻滞在有丝分裂中期。常规收获后制片，Earle's显带液87.5 °C水浴显带，吉姆萨染液染色，完成R显带。通过Ikaros系统（德国Metasystems公司产品）全自动扫描并进行分析，每个标本分析20个中期分裂象，核型描述依据《人类细胞基因组学国际命名体制（ISCN2016）》。

3. 基因突变分析：收集患者全骨髓细胞，使用厦门至善核酸提取仪器（型号824s）以及核酸提取试剂（货号 606001）提取患者基因组DNA。使用M220聚焦超声波破碎仪（美国Covaris公司产品）超声打断基因组DNA至长度约220 bp。片段产物采用纳昂达（南京）生物科技有限公司NadPrep DNA通用型文库构建试剂盒（货号1002103）探针靶向捕获建库，NextSeq550测序仪（美国因美纳公司产品）测序，平均测序深度1 000×。FLT3-ITD突变采用毛细管电泳方法检测。采用实验室自建方法检测分析髓系肿瘤常见52种基因突变[9]。

4. 疗效判定：在MDS和MDS/MPN患者中，骨髓或外周血中原始细胞比例≥20％定义为疾病进展为急性白血病。总生存（OS）期定义为患者自确诊至死亡或末次随访的时间。无进展生存期定义为患者自确诊至出现疾病进展、死亡或末次随访的时间。AML患者治疗方案见本中心既往报道[10]。

5. 随访：通过查阅住院系统电子病历及电话随访的方式进行随访，随访截止时间为2026年4月。

6. 统计学处理：应用SPSS 26软件进行统计学分析。定性资料用例数（构成比）表示，定量资料用*M*（范围）表示。连续变量采用非参数Mann-Whitney *U*检验，分类变量的组间比较采用卡方检验或Fisher精确概率法。生存分析采用Kaplan-Meier法。*P*<0.05为差异有统计学意义。

## 结果

1. 临床特征：183例SF3B1突变髓系肿瘤患者中位年龄62（16～83）岁，男107例（58.5％），女76例（41.5％）。根据WHO造血和淋巴组织肿瘤分型指南第5版标准，CH（包括潜能未定克隆性造血和意义未明克隆性血细胞减少）患者10例（5.5％），MPN患者7例（3.8％），MDS患者106例（58.0％），MDS/MPN患者27例（14.8％），AML患者33例（18.0％）（**扫描本文首页二维码查阅附图1**）。MDS-SF3B1与伴SF3B1突变的其他髓系肿瘤患者的临床特征见附表1（**扫描本文首页二维码查阅**）。共检测到31种SF3B1基因突变类型，突变位点主要分布于SF3B1基因HEAT结构域第581～933位氨基酸。其中MPN患者SF3B1突变均发生在第666位氨基酸（附图2，**扫描本文首页二维码查阅**）。伴SF3B1突变的MDS患者中位基因突变数为2（1～7），低于伴SF3B1突变的MDS/MPN患者［4（1～9）］和AML患者［3（1～9）］，差异均有统计学意义（*P*值均<0.001）。共突变发生率≥5％的基因主要有ASXL1、TET2、RUNX1、DNMT3A、BCOR、EZH2、GATA2、JAK2、NRAS、KRAS和FLT3等。另有6.6％（12/183）患者携带MECOM（染色体3q26.2）重排（图1）。

**图1:**
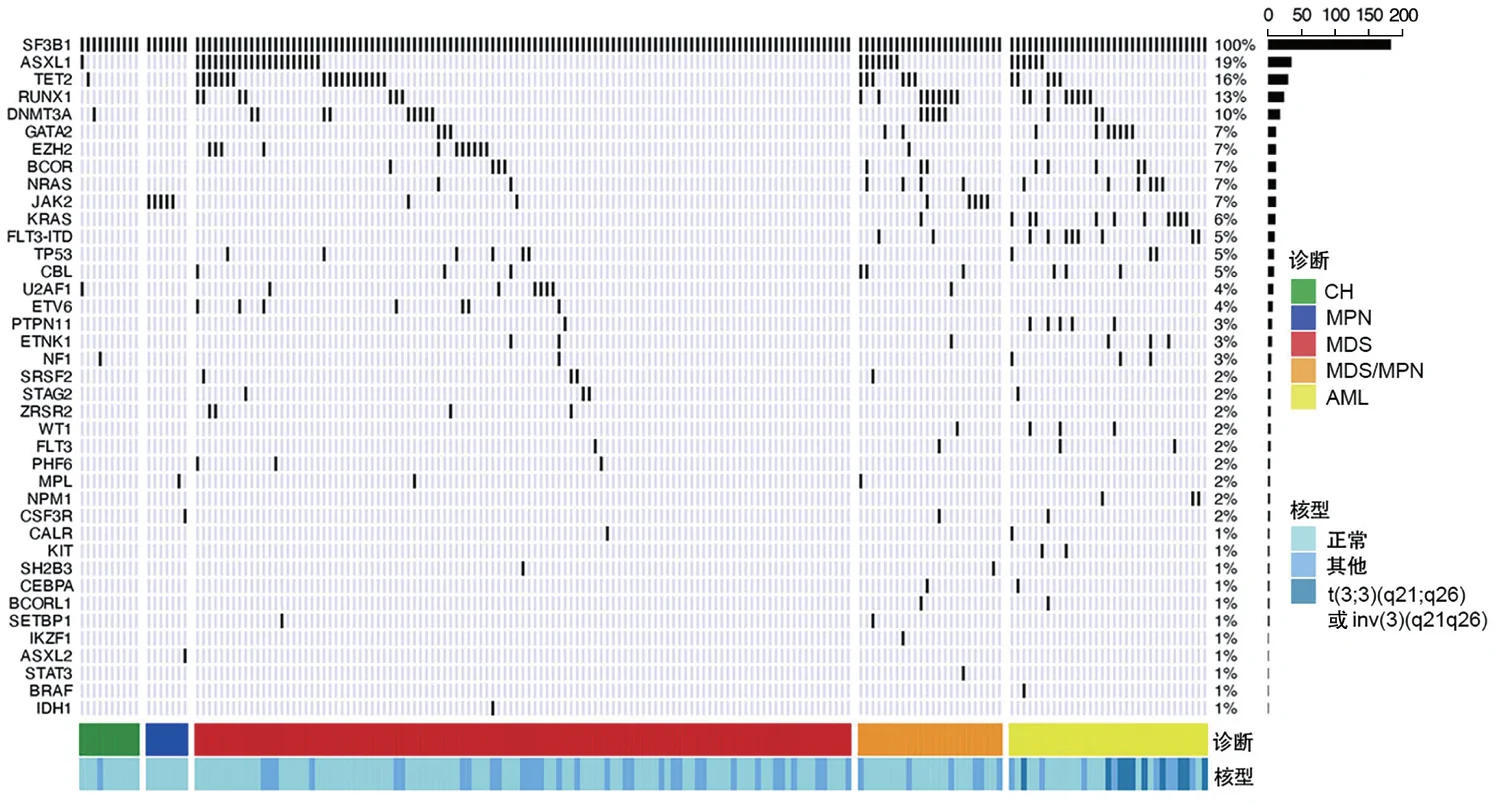
183例伴SF3B1基因突变髓系肿瘤患者的突变谱 **注** CH：克隆性造血；MPN：骨髓增殖性肿瘤；MDS：骨髓增生异常肿瘤；MDS/MPN：骨髓增生异常肿瘤/骨髓增殖性肿瘤；AML：急性髓系白血病

2. SF3B1突变的变异等位基因频率（variant allele frequency，VAF）：CH患者SF3B1突变VAF中位数为3.6％（2.0％～19.7％），显著低于MPN患者的41.8％（2.0％～48.0％）、MDS患者的34.3％（2.0％～50.8％）、MDS/MPN患者的43.8％（5.1％～66.0％）、AML患者的40.6％（2.9％～50.0％），差异有统计学意义（*P*值均<0.001）。在103例CH和骨髓原始细胞比例<5％的MDS患者中，21例患者SF3B1突变VAF<10％，82例患者SF3B1突变VAF≥10％。SF3B1突变VAF<10％的患者HGB高于SF3B1突变VAF≥10％的患者［95（59～209）g/L对76（26～117）g/L，*P*＝0.002］。SF3B1突变VAF<10％的患者环状铁粒幼红细胞比例低于SF3B1突变VAF≥10％的患者［3（0～64）％对41（0～86）％，*P*<0.001］。所有骨髓环状铁粒幼红细胞比例≥5％的患者SF3B1突变VAF均高于5％。在SF3B1突变VAF≥10％的患者中，1例意义未明克隆性血细胞减少（clonal cytopenia of undetermined significance，CCUS）患者和3例MDS伴低原始细胞（MDS-LB）患者骨髓环状铁粒幼红细胞比例低于5％；另有3例MDS-SF3B1患者骨髓涂片瑞氏染色未观察到细胞发育异常，但是铁染色提示环状铁粒幼红细胞比例≥5％。此外，在21例SF3B1突变VAF<10％的患者中，11例SF3B1突变VAF<5％的患者骨髓环状铁粒幼红细胞比例均低于5％；另10例患者中有2例CCUS和1例MDS-LB患者骨髓环状铁粒幼红细胞比例低于5％。在SF3B1突变VAF<10％的4例MDS-LB患者中均检测到额外染色体异常。在骨髓原始细胞比例<5％的MDS患者中，SF3B1突变VAF≥10％的患者2年OS率优于SF3B1突变VAF<10％患者（98.1％对74.1％，*P*＝0.008）。在MDS-SF3B1患者中，SF3B1突变VAF≥10％与VAF<10％的患者2年OS率的差异无统计学意义（98.1％对100％，*P*＝0.319）。

3. JAK2 V617F突变的发生率及VAF：在183例患者中，6.6％（12/183）合并JAK2 V617F突变。JAK2 V617F突变在MPN和MDS/MPN中的发生率分别为71.4％（5/7）和22.2％（6/27），均高于在MDS中的发生率1.2％（1/84），差异有统计学意义（*P*值分别为<0.001和0.001）。此外，在1例MDS患者中，JAK2突变的VAF（7.1％）低于SF3B1突变的VAF（22.6％）。5例MPN患者JAK2突变的VAF与SF3B1突变的VAF差异无统计学意义［39.5％（30.0％～89.4％）对41.8％（2.0％～46.9％），*P*＝0.917］。但其中1例MPN患者JAK2突变的VAF（31.0％）高于SF3B1突变的VAF（2％）。

4. RUNX1突变的发生率：13.1％（24/183）患者合并RUNX1突变。MDS/MPN和AML患者RUNX1突变的发生率分别为33.3％（9/27）和24.2％（8/33），均高于MDS患者［6.6％（7/106）］，差异有统计学意义（*P*值分别为0.002和0.008）。骨髓原始细胞比例<5％的MDS患者中，RUNX1突变组和野生型组MDS患者的骨髓原始细胞比例分别为3％（0.5％～4.0％）和1.0％（0％～4.0％），差异有统计学意义（*P*＝0.028）。在24例SF3B1合并RUNX1突变髓系肿瘤患者中，8例AML中6例为初诊AML，1例为MDS/MPN进展为AML，1例为MDS伴高原始细胞（MDS-IB）进展为AML。另外16例MDS/MPN和MDS患者中，3例MDS/MPN后期进展为AML，1例MDS-IB后期进展为AML。在MDS和MDS/MPN患者中，伴RUNX1突变的患者具有更高的进展为AML的风险（*P*<0.001）（图2）。此外，5例伴RUNX1突变MDS-SF3B1患者中，2例分别在2个月和8个月后进展为MDS-IB。另有1例MDS-SF3B1患者确诊16个月后进展为AML，进展前1个月在骨髓标本中检测到新发RUNX1突变。

**图2:**
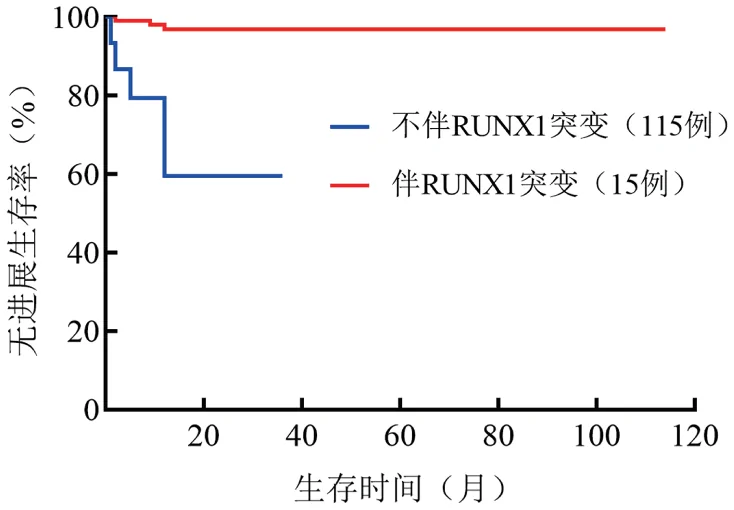
伴和不伴RUNX1突变的MDS和MDS/MPN患者的无进展生存曲线 **注** MDS：骨髓增生异常肿瘤；MDS/MPN：骨髓增生异常肿瘤/骨髓增殖性肿瘤

5. RAS通路相关基因突变的发生率：6.0％（11/183）患者合并KRAS突变，其中10例为AML，占AML患者的30.3％（10/33）；另有1例为慢性粒-单核细胞白血病（chronic myelomonocytic leukaemia，CMML）。AML和CMML患者RAS通路相关基因（KRAS、NRAS、CBL和PTPN11）突变的发生率分别为66.7％（22/33）和38.5％（5/13），均高于MDS患者（3.8％，4/106），差异均有统计学意义（*P*值分别为<0.001和0.008）。

6. 生存分析：比较MDS-SF3B1、MDS/MPN和AML患者的预后，MDS-SF3B1（91.6％）和MDS/MPN（79.9％）患者的5年OS率优于AML（63.8％）患者（*P*值分别为<0.001、0.033）（图3），MDS-SF3B1和MDS/MPN患者的5年OS率差异无统计学意义（91.6％对79.9％，*P*＝0.200）（图3）。在AML中，造血干细胞移植患者2年OS率与化疗组比较差异有统计学意义（80.0％对12.5％，*P*<0.001）。

**图3:**
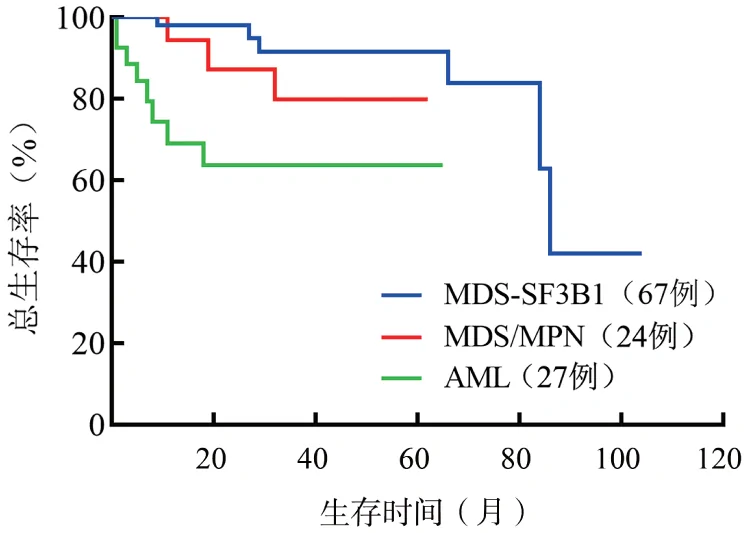
伴SF3B1突变MDS-SF3B1、MDS/MPN和AML患者的总生存曲线 **注** MDS-SF3B1：MDS伴低原始细胞和SF3B1突变；MDS/MPN：骨髓增生异常肿瘤/骨髓增殖性肿瘤；AML：急性髓系白血病

## 讨论

本研究回顾性分析了183例SF3B1突变髓系肿瘤患者，共检测到31种SF3B1突变类型，突变均发生在HEAT区，最常见的突变类型发生在第700、666和625位氨基酸，与国内外报道相符[11]–[12]。根据WHO第5版造血和淋巴组织肿瘤分型指南诊断标准，本研究各类型疾病所占比例从高到低依次为MDS、AML、MDS/MPN、CH和MPN，不同疾病患者携带的基因突变数量存在差异，提示不同疾病患者具有不同的突变谱特征。

在CH和骨髓原始细胞比例<5％的MDS患者中，SF3B1突变VAF≥10％患者的HGB显著低于SF3B1突变VAF<10％患者。SF3B1突变能通过诱导MAP3K7转录本剪接错误下调MAP3K7表达，促进晚期幼红细胞凋亡，导致SF3B1突变患者贫血[13]。SF3B1突变VAF≥10％患者骨髓环状铁粒幼红细胞比例显著高于SF3B1突变VAF<10％患者。SF3B1基因突变能阻断铁从线粒体转运到胞质，使铁在线粒体内堆积并促进环状铁粒幼红细胞形成[14]。上述结果提示，在CH和骨髓原始细胞比例<5％的MDS患者中，SF3B1突变的VAF与患者HGB水平和环状铁粒幼红细胞比例密切相关。因此，SF3B1突变VAF对于MDS-SF3B1与CH的鉴别诊断具有潜在临床指导意义。WHO第5版分型指南将MDS-SF3B1定义为独立亚型，诊断标准为伴有SF3B1突变且环状铁粒幼红细胞比例≥5％[1]。在CH和骨髓原始细胞比例<5％的MDS患者中，环状铁粒幼红细胞比例≥5％患者的SF3B1突变VAF均高于5％，SF3B1突变VAF<5％患者的环状铁粒幼红细胞比例均<5％。这一结果也与WHO第5版指南中“SF3B1突变VAF<5％不支持MDS-SF3B1诊断”的建议相符。

在国际共识分类（international consensus classification，ICC）指南中，当患者SF3B1突变VAF≥10％时可以不考虑骨髓细胞发育异常及环状铁粒幼红细胞比例，直接诊断为MDS-SF3B1[15]。本研究SF3B1突变VAF≥10％的CH和骨髓原始细胞比例<5％的MDS患者中，3例SF3B1突变VAF≥10％患者骨髓细胞瑞氏染色未观察到发育异常，但铁染色提示骨髓环状铁粒幼红细胞比例≥5％。这一现象支持ICC指南的诊断标准。另有1例CCUS和3例MDS-LB患者SF3B1突变VAF≥10％，4例患者骨髓环状铁粒幼红细胞比例<5％。根据ICC指南，这4例患者符合MDS-SF3B1诊断。生存预后分析结果提示，在骨髓原始细胞比例<5％的MDS患者中，SF3B1突变VAF≥10％的MDS患者的OS优于SF3B1突变VAF<10％的MDS患者，与国内报道相符[16]。在本研究根据WHO第5版指南诊断的MDS-SF3B1患者中，SF3B1突变VAF≥10％患者与VAF<10％患者的预后差异无统计学意义。SF3B1突变的MDS-LB患者均存在额外染色体异常，可能是上述两组生存预后分析结果差异的原因之一。上述生存预后分析结果与ICC指南将SF3B1突变VAF≥10％的MDS患者直接诊断为预后良好的MDS-SF3B1相符，也与WHO第5版指南将SF3B1突变VAF≥5％和环状铁粒幼红细胞比例≥5％的患者纳入预后良好MDS-SF3B1相符。骨髓原始细胞比例<5％且SF3B1突变VAF<10％的患者具有诊断为CCUS、MDS-LB和MDS-SF3B1的可能。因此，骨髓细胞涂片铁染色检查和骨髓染色体核型分析对MDS-SF3B1的诊断有重要价值，尤其是在SF3B1突变VAF<10％的患者中。

ICC指南对MDS-SF3B1诊断还要求排除RUNX1突变。在本研究伴SF3B1突变髓系肿瘤患者中，RUNX1突变在MDS/MPN和AML中的发生率显著高于MDS。在MDS和MDS/MPN中，携带RUNX1突变的患者有高风险进展为AML，与国内外报道相符[16]–[17]。在根据WHO第5版诊断的MDS-SF3B1患者中，携带RUNX1突变的患者骨髓原始细胞比例较高，并可能向MDS-IB和AML进展。因本研究中RUNX1突变的MDS-SF3B1患者例数较少，RUNX1突变能否影响MDS-SF3B1患者的OS还有待进一步积累病例。

JAK2突变在MPN和MDS/MPN中的发生率显著高于MDS。SF3B1突变可以与MPN相关JAK2、CALR和MPL等基因突变共存[18]–[19]。在MPN中，SF3B1突变均发生在第666位氨基酸，提示JAK2 V617F突变和SF3B1基因K666位点突变可能具有协同致病机制。MPN中JAK2突变的VAF常不低于SF3B1突变的VAF，且有1例患者JAK2突变的VAF显著高于SF3B1突变的VAF。而在MDS中SF3B1突变的VAF高于JAK2突变的VAF。提示在伴SF3B1突变的MPN患者中，JAK2突变可能是主要克隆事件，而在伴SF3B1突变的MDS患者中，JAK2突变可能是次要克隆事件。此外，信号分子RAS通路相关基因突变在CMML和AML中的发生率显著高于MDS。RAS通路相关基因是白血病发病较晚期的突变事件，RAS通路相关突变与原始细胞比例升高密切相关[20]。

因此，在伴SF3B1突变的髓系肿瘤患者中，额外协同突变（如RUNX1、JAK2、RAS家族突变等）对于疾病的诊断有潜在指导价值。在伴SF3B1突变的AML中，除RUNX1突变和RAS通路相关基因突变以外，还常携带AML常见重现性遗传学异常MECOM重排[21]。SF3B1突变能够与MECOM重排协同，导致EVI1基因异常剪接并增强细胞自我更新能力，从而推动恶性克隆细胞向AML进展[22]。因此，WHO第5版指南将髓系肿瘤伴MECOM重排归为AML伴MECOM重排亚型。在本研究中，MDS-SF3B1患者的预后优于伴SF3B1突变的MDS/MPN患者，伴SF3B1突变AML患者预后最差。造血干细胞移植能显著改善伴SF3B1突变AML患者的预后。未来随着SF3B1突变靶向药物的研发，预后较差的SF3B1突变血液肿瘤患者可能有更多的治疗手段[23]。

本研究存在一定的局限性，作为单中心回顾性研究，样本量较小，可能存在选择偏倚，结论有待多中心、大样本研究进一步验证。

综上所述，在伴SF3B1突变髓系肿瘤中，不同SF3B1突变髓系肿瘤患者具有不同的突变谱特征，SF3B1突变VAF和协同突变类型对于患者的诊断具有潜在的临床指导意义。

## Supplementary Material


